# Anthropometric﻿ accuracy of three-dimensional average faces compared to conventional facial measurements

**DOI:** 10.1038/s41598-021-91579-4

**Published:** 2021-06-10

**Authors:** Zhiyi Shan, Richard Tai-Chiu Hsung, Congyi Zhang, Juanjuan Ji, Wing Shan Choi, Wenping Wang, Yanqi Yang, Min Gu, Balvinder S. Khambay

**Affiliations:** 1 Orthodontics, Division of Paediatric Dentistry and Orthodontics, Faculty of Dentistry, The University of Hong Kong, Hong Kong, SAR China; 2 Department of Computer Science, Chu Hai College of Higher Education, Hong Kong, SAR China; 3 Discipline of Oral and Maxillofacial Surgery, Faculty of Dentistry, The University of Hong Kong, Hong Kong, SAR China; 4 Department of Computer Science, The University of Hong Kong, Hong Kong, SAR China; 5 Department of Stomatology, Second People’s Hospital of Yunnan Province, Kunming, People’s Republic of China; 6 Texas A&M University, Texas, USA; 7 Institute of Clinical Sciences, College of Medical and Dental Sciences, The School of Dentistry, University of Birmingham, Birmingham, UK

**Keywords:** Anatomy, Medical research, Engineering

## Abstract

This study aimed to evaluate and compare the accuracy of average faces constructed by different methods. Original three-dimensional facial images of 26 adults in Chinese ethnicity were imported into Di3DView and MorphAnalyser for image processing. Six average faces (Ave_D15, Ave_D24, Ave_MG15, Ave_MG24, Ave_MO15, Ave_MO24) were constructed using “surface-based registration” method with different number of landmarks and template meshes. Topographic analysis was performed, and the accuracy of six average faces was assessed by linear and angular parameters in correspondence with arithmetic means calculated from individual original images. Among the six average faces constructed by the two systems, Ave_MG15 had the highest accuracy in comparison with the conventional method, while Ave_D15 had the least accuracy. Other average faces were comparable regarding the number of discrepant parameters with clinical significance. However, marginal and non-registered areas were the most inaccurate regions using Di3DView. For MorphAnalyser, the type of template mesh had an effect on the accuracy of the final 3D average face, but additional landmarks did not improve the accuracy. This study highlights the importance of validating software packages and determining the degree of accuracy, as well as the variables which may affect the result.

## Introduction

Anthropometric facial analysis refers to the quantitative evaluation of human facial morphology, and is essential in multiple clinical disciplines, including paediatrics, orthodontics, and craniofacial surgery^1–4^. Over the past decades, databases of anthropometric facial norms covering both size and form have been established for over 25 ethnicities^5^. The traditional approach of establishing anthropometric normal values has been to select the population of interest and then determine a series of average linear Euclidian distances and angular measurements, based on historical requirements. These measurements can be derived by direct clinical measurements^2^ or measurements from conventional photographs^6^ or cephalograms^7^. It is common practice, based on these methods, to only extract a limited number of landmarks and measurements to represent the facial complex. These measurements were appropriate at the time, using the technology available. As technology has advanced more complex but routine methods of 3D facial capture are available^8, 9^.

Previous studies have reported the use of “3D average facial images” as an anthropometric tool in facial analysis^10–14^. However studies do not generally use the same methodology to create the 3D average facial meshes. In a recent study, average facial meshes have been used to develop normative average 3D faces of healthy infants to describe normative longitudinal average 3D facial growth in infants^10^. The study created the average facial mesh by non-rigid deformation of a generic mesh template using the Coherent Point Drift algorithm. Followed by application of the ray casting algorithm to create a uniform mesh pattern for all subjects, with the same number of vertices, and from this creating an 3D average face. Other studies have used average faces to compare different population groups^11^. The average faces were generated using an image pre-alignment pipeline and the “built-in algorithm” in RapidForm software (Geomagic Korea, Seoul Korea) to determine the “best fit” of the facial images and then averaging the 3D datapoints of the images, based on a facial template. Average faces have been also used to analyse facial soft tissue following orthognathic surgery^12^. Using “in-house” developed software two 3D average faces were produced, pre-surgery and post-surgery, these were then used to assess surgical changes in the x, y and z direction. These 3D average faces were produced by “averaging” the indices of the conformed generic mesh to calculate where each of the corresponding vertices (those that share the same index value) were likely to be, across the facial meshes of the whole sample. Then using dense correspondence analysis to create a 3D average face. An alternative software solution for generating an average 3D facial mesh is the use of MorphAnalyser, which has been used to assess cleft outcome in adults^13^ and infants^14^. MorphAnalyser again uses a base mesh and dense correspondence to create an average 3D facial mesh template. All these methods of 3D average face generation rely on various computer algorithms and processes, which as clinicians we assume to be valid and have an acceptable level of accuracy.

However, no study has investigated the validity or accuracy of 3D average faces in comparison to the conventional anthropometric methods by which previous and current 2D facial norms are calculated. Therefore, the aim of this study was to assess the accuracy of linear and angular measurements obtained using conventional digital anthropometry, with the “3D average faces” produced by two different software packages; Di3DView (Di4D SNAP, Dimensional Imaging Ltd., Hillington, Glasgow, UK) and MorphAnalyser (http://cherry.dcs.aber.ac.uk/morphanalyser). In addition, differences in the surface topography of the 3D average faces were compared.

## Material and methods

### Sample size calculation

Based on a previous study^15^, the maximum system error reported during image conformation using Di3DView was 0.53 ± 0.62 mm. Following a sample size calculation a minimum of 26 individuals would be required to achieve a significance level of 0.05 and power of 0.95.

### Ethical approval

This retrospective study was performed in the Department of Orthodontics, Faculty of Dentistry, the University of Hong Kong. Ethical approval was obtained from the Institutional Review Board (IRB) of the University of Hong Kong and Hong Kong Hospital Authority, Hong Kong West Cluster (UW18-079). IRB approved the need of waived informed consent. All methods were carried out in accordance with relevant guidelines and regulations.

### Sample selection

The sample consisted of 26 Chinese adult patients (15 Male and 11 Female) who had attended the Department of Orthodontics for routine orthodontic treatment and had 3D facial images taken as part of their routine records. The inclusion criteria was a follows:

- Males with no facial hair,
- Images of the full face including the forehead was visible,
- No facial scarring.

### Image capture

Static three-dimensional (3D) images of each participant were taken using a 3dMDface System (3dMD Inc., Atlanta, GA, USA) by one professional photographer. The accuracy of the system had been previously published and was reported to be lower than 0.2 mm root mean square (RMS)^16, 17^. Prior to image capture, the 3dMDface system was calibrated according to the manufacturers instructions. Immediately prior to capturing the 3D image, participants were seated 100 cm away from the capture system, looking forward with Frankfort plane parallel to the floor, and any glasses and jewellery removed. The camera system captured six 2D images; four black and white pictures, depicting facial structures and spatial relationships to form a facial framework; two coloured images to project the texture information onto mesh framework^16^. The capture took 1.5 ms and were saved as an object wavefront file (.OBJ) for later analysis.

### Landmarking and facial image conformation

The process of average face construction involved two steps:

- Step 1—Conformation, which involved transforming the topography of a generic mesh to each individuals original facial mesh. The generic mesh was a computer generated symmetrical facial image constructed of 3763 vertices. This required corresponding landmarks to be placed on each original facial mesh and the generic mesh. Using these landmarks as “anchors”, the remaining generic mesh was elasticity deformed to fit the remaining original facial surface. As a result, the generic mesh had the same surface topography of the patients face but the number of vertices was the same across every face and listed or indexed in the same order to maintain anatomical correspondence. The generic mesh was of lower resolution than the original 3D captured facial mesh.
- Step 2—Constructing the average facial surface.

### Process for Di3DView

**Figure 1:**
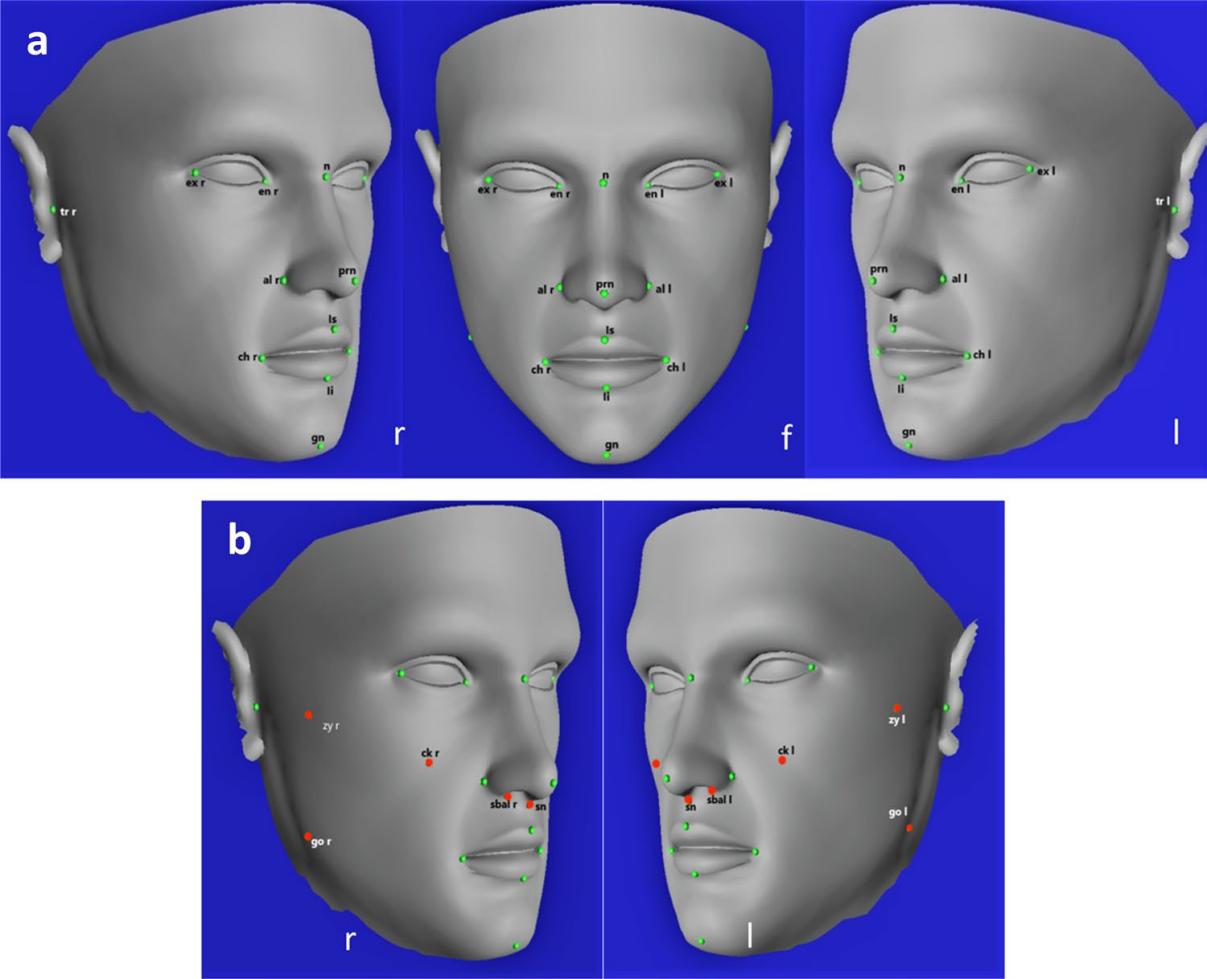
Generic mesh in Di3DViewViewer with registration landmarks (**a)** 15 landmarks for conformation in Di3DView and MorphAnalyser; (**b)** extra 9 landmarks for conformation in Di3DView.

For each patient the original 3D facial image in .OBJ format was imported into Di3DView. Fifteen landmarks were digitized on the original 3D facial image and saved in .OBJ format, Fig. 1a. For each image nine additional landmarks were digitized closer to the periphery of the original 3D facial image and again saved in .OBJ format, Fig. 1b. Detailed definition of all landmarks are illustrated in Table 1. The same 15 and additional 9 landmarks were digitized on the generic mesh and saved for the conformation process (24 landmarks in total).

**Table 1:** The definition of registered landmarks for conformation and/or measurement.

Types			Landmarks	Abbreviation	Type	Definition
15 for conformation	24 for conformation	28 for measurement	Exocanthion	Ex	Bilateral	Most lateral point of the palpebral fissure, at the outer commissure of the eye
			Endocanthion	En	Bilateral	Most medial point of the palpebral fissure, at the inner commissure of the eye
			Nasion	N	Medial	Point directly anterior to the nasofrontal suture, in the midline, overlying n
			Alare	Al	Bilateral	The most lateral point on the nasal alare
			Pronasale	Prn	Medial	The most protruded point of the apex nasi
			Cheilion	Ch	Bilateral	Outer corners of the mouth where the outer edges of the upper and lower vermilions meet
			Labrale Superius	Ls	Medial	The midpoint of the upper vermillion line
			Labrale Inferius	Li	Medial	The midpoint of the lower vermillion line
			Gnathion	Gn	Medial	Median point halfway between pog and Me
			Tragus	Tr	Bilateral	The prominence on the inner side of the external ear, in front of and partly closing the passage to the organs of hearing
			Gonion	Go	Bilateral	Most lateral point on the mandibular angle
			Zygion	Zy	Bilateral	Most lateral point overlying each zygomatic arch, identified as the point of maximum bizygomatic breadth of the face
			Subnasale	Sn	Medial	Median point at the junction between the lower border of the nasal septum and the philtrum area
			Subalare	Sbal	Bilateral	The point on the lower margin of the base of the nasal ala where the ala disappears into the upper lip skin
			Cheek	Ck	Bilateral	The intersection point of the lines connecting al-tr and ex-ch
			Christa Philtri	Cph	Bilateral	Point on each elevated margin of upper lip at the junction of vermillion line of upper lip and white roll line
			Supermentale	Sm	Medial	Deepest midline point of the mentolabial sulcus
			Pogonion	Pog	Medial	The most prominent point of the chin

For conformation, the generic mesh and its digitised 15 landmarks were imported into Di3DView together with each individual’s original facial 3D image and corresponding digitized 15 landmarks. Using the “Shape transfer function”, the generic mesh was elasticity deformed to fit the remaining original facial surface. The new “conformed generic mesh” was saved again in OBJ format (Di3D_15). This process was repeated using the 24 landmarks (Di3D_24). As a result, two conformed generic meshes were produced, one based on 15 landmarks and the other using 24 landmarks, these would be used for the average face construction.

### Process for MorphAnalyser

For each patient the original 3D facial image in .OBJ format (high resolution) was imported into MorphAnalyser Version 2.4 (http://cherry.dcs.aber.ac.uk/morphanalyser).

MorphAnalyser did not routinely use a generic mesh for conformation, but instead used a “standard” template. In this case, the standard template or mesh was made up of 39,256 vertices and was chosen from one randomly selected original facial image. Conformation created the same mesh structure for all the images by warping all images to one image’s structure i.e. all images were warp to the “standard template”. Following landmark digitization as previously mentioned, the “conformation” process was conducted by elastically deforming or warping the template onto each individual facial surface.

To determine the effect of template mesh (“standard” template based on one randomly selected original facial image versus generic mesh) on conformation and averaging, the same generic mesh used in the Di3DView process was used in MorphAnalyser with digitation of both 15 and 24 landmarks. As a result, four conformed meshes were generated, two based on a randomly selected original facial image (Morph_Original_15 and Morph_Original_24) and the other two using the generic mesh (Morph_Generic_15 and Morph_Generic_24). For each patient both meshes were saved in .OBJ format for average face construction.

### Average face construction

#### Di3DView

**Figure 2:**
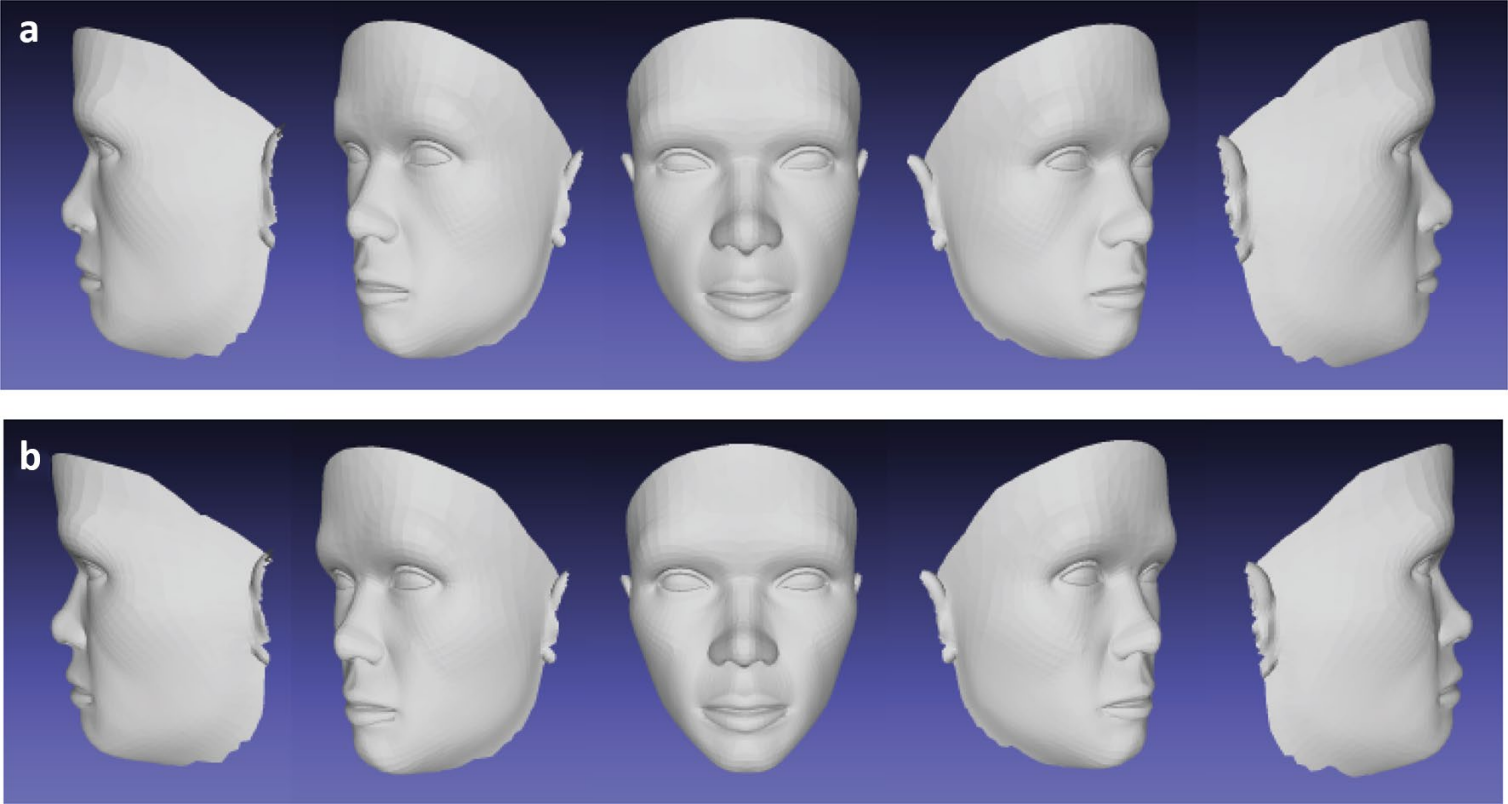
Average faces of 26 subjects generated by Di3DView with 15 and 24 landmark digitation. (**a)** Ave_D15; (**b)** Ave_D24.

All the conformed generic images (Di3D_15) based on the 15 landmarks were saved in a single folder. These were then used in the “Average Face” function in Di3DView to create an average 3D facial mesh surface (Ave_D15) based on the mean position of each correspondence of all individuals. This was saved in .OBJ format. The same process was used for the conformed generic images (Di3D_24) based on the 24 landmarks. The average facial 3D mesh surface produced (Ave_D24) was again saved in .OBJ format, Fig. 2.

#### MorphAnalyser

The first conformed generic mesh image from the Morph_Original series was loaded into MorphAnalyser using the “Add to Average…" with two sets of landmark registration. The next patient’s image was added to the average, until all the Morph_Original files have been included. The resulting average images (Ave_ MO15 and Ave_MO24) were saved in .OBJ format. This process was repeated for the Morph_Generic series of images, resulting in a further two new average 3D facial surfaces (Ave_ MG15 and Ave_MG24), again saved in .OBJ format.

**Figure 3:**
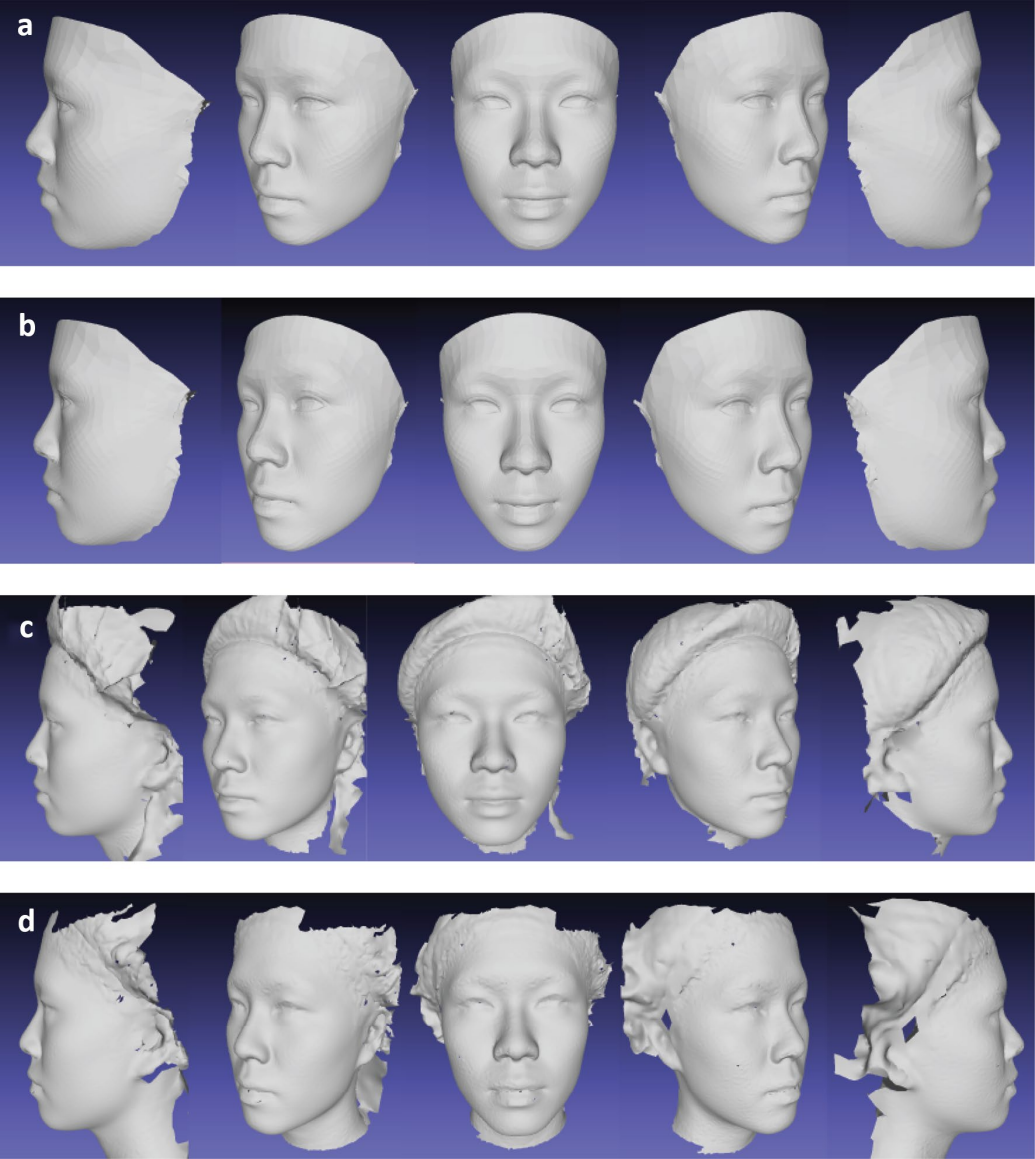
Average faces of 26 subjects generated by MorphAnalyser with two sets of landmarks on one randomly-selected original image and the generic mesh respectively. (**a)** Ave_MG15, (**b)** Ave_MG24, (**c)** Ave_MO15, (**d)** Ave_MO24.

This resulted in six average facial images in total, two produced by Di3DView based on 15 and 24 landmarks, and four produced by MorphAnalyser based on template density difference (“standard” template based on one randomly selected original facial image versus generic mesh) and landmark variation 15 landmarks versus 24 landmarks), Fig. 3.

### Analysis

#### Topographic analysis of average faces

The four average faces (Ave_D15, Ave_D24, Ave_MG15, Ave_MG24) were imported into MATLAB software (Version 9.7.0. Natick, Massachusetts, USA) for surface topography analysis. In-house software was developed to measure the Median Euclidean Distance (MED) between the four different average facial mesh combinations following superimposition on the centroids of each of the average facial meshes. The MED is a measure of the distance between corresponding vertices between any two facial meshes. In order for this to work all the facial meshes had to have the same number of vertices i.e. in this case the made up from the same generic mesh. This meant that the meshes derived on the randomly selected original facial image, using MorphAnalyser were not included (Ave_ MO15 and Ave_MO24). This was because they were made of a different number of vertices and so correspondences, and therefore measurements, with the generic mesh could not be calculated. The Median of Euclidean Distances (MED) were tested for normality and compared using a one-sample Wilcoxon signed test to a hypothesis median of 2 mm, as this was determined to be clinically significant.

#### Anthropometric assessment

For each patient seventeen linear (L) and seven angular (A) measurements were taken of their static three-dimensional (3D) facial image using Di3DView, Table 1. This was repeated for all patients and a mean value obtained for each of the 24 measurements; these were taken as the “gold standard”.

To determine the validity of each of the average faces, the gold standard anthropometric measurements were compared to the measurements obtained from each of the six average faces. The 24 measurements were taken when 15 landmarks were used for conformation (L1/A1) and again where 24 landmarks were used for conformation (L2/A2), using Di3DView and MorphAnalyzer. In both cases additional landmarks were used in the measurements that were not used during the conformation process.

The level of statistical significance was set at 0.05. In addition, clinical significance for linear parameters was 2 mm, and that for angular measurements as 5°. The statistical analysis was conducted using Statistical Package for Social Sciences V.25 (SPSS Inc., Chicago, Illinois, USA).

#### Error study

To assess intra-operator error, linear and angular measurement were undertaken twice with 2-week interval on the original images and generated average faces by one experienced examiners (SZY). For anthropometric assessment, the differences in landmark coordinates between the first and second digitisation were used for analysis of the errors of the assessment method. Systematic error was assessed by using a paired sample t-tests (p-values) and random error assessed using correlation coefficients, Table 2**.**

**Table 2:** Each of the four images (average faces) were landmarked twice, 2 weeks apart, and the differences in landmark coordinates between the first and second digitisation were used for analysis of the errors of the method.

	Ave_D15			Ave_D24			Ave_MG15			Ave_MG24			Ave_MO15			Ave_MO24		
	X	Y	Z	X	Y	Z	X	Y	Z	X	Y	Z	X	Y	Z	X	Y	Z
Ex (R)	0.3	0.0	0.1	0.0	0.0	0.0	0.0	0.2	0.2	0.1	0.0	0.1	0.5	0.0	0.1	0.2	0.1	0.4
En (R)	0.1	0.2	0.3	0.1	0.3	0.3	0.0	0.3	0.1	0.1	0.0	0.0	0.1	0.0	0.2	0.2	0.1	0.1
En (L)	0.1	0.1	0.1	0.0	0.0	0.0	0.0	0.0	0.1	0.0	0.0	0.1	0.2	0.0	0.0	0.3	0.1	0.1
Ex (L)	0.4	0.1	0.2	0.6	0.1	0.1	0.0	0.1	0.1	0.2	0.1	0.0	0.4	0.1	0.3	0.3	0.0	0.2
N	0.0	0.1	0.1	0.2	0.0	0.1	0.1	0.0	0.1	0.2	0.0	0.5	0.1	0.0	0.2	0.4	0.1	0.0
Al (R)	0.1	0.1	0.3	0.0	0.1	0.2	0.0	0.1	0.4	0.0	0.1	0.2	0.0	0.1	0.2	0.1	0.0	0.4
Al (L)	0.2	0.2	0.2	0.1	0.1	0.0	0.2	0.2	0.0	0.0	0.1	0.1	0.0	0.0	0.3	0.1	0.3	0.2
Prn	0.1	0.1	0.2	0.1	0.0	0.4	0.0	0.0	0.1	0.0	0.1	0.1	0.2	0.0	0.2	0.1	0.1	0.1
Sn	0.0	0.2	0.3	0.1	0.0	0.1	0.3	0.1	0.1	0.1	0.0	0.1	0.2	0.0	0.1	0.2	0.1	0.0
Sbal (R)	0.1	0.1	0.1	0.1	0.1	0.1	0.0	0.2	0.2	0.1	0.0	0.0	0.2	0.0	0.1	0.1	0.0	0.0
Sbal (L)	0.0	0.1	0.0	0.1	0.0	0.0	0.1	0.1	0.0	0.1	0.1	0.2	0.1	0.1	0.2	0.3	0.1	0.2
Ch (R)	0.0	0.0	0.2	0.1	0.0	0.1	0.1	0.2	0.2	0.2	0.1	0.0	0.0	0.0	0.1	0.1	0.1	0.1
Ch (L)	0.4	0.1	0.1	0.1	0.1	0.1	0.1	0.1	0.1	0.3	0.0	0.1	0.2	0.0	0.0	0.4	0.0	0.0
Cph (R)	0.0	0.0	0.0	0.1	0.0	0.2	0.0	0.0	0.2	0.2	0.1	0.1	0.3	0.0	0.1	0.3	0.0	0.1
Cph (L)	0.1	0.0	0.1	0.5	0.1	0.2	0.0	0.0	0.3	0.2	0.2	0.1	0.2	0.0	0.2	0.4	0.1	0.0
Ls	0.1	0.1	0.1	0.1	0.0	0.0	0.2	0.0	0.1	0.1	0.1	0.3	0.3	0.0	0.1	0.0	0.0	0.0
Li	0.0	0.1	0.1	0.0	0.1	0.1	0.1	0.0	0.0	0.0	0.0	0.0	0.1	0.2	0.4	0.1	0.2	0.2
Gn	0.0	0.1	0.1	0.0	0.3	0.4	0.1	0.0	0.0	0.0	0.6	0.3	0.1	0.0	0.1	0.0	0.1	0.1
Sm	0.0	0.0	0.0	0.1	0.0	0.1	0.1	0.1	0.2	0.0	0.1	0.2	0.0	0.1	0.2	0.4	0.0	0.1
Pog	0.0	0.0	0.2	0.1	0.0	0.1	0.0	0.0	0.1	0.0	0.0	0.2	0.1	0.0	0.1	0.3	0.0	0.1
Go (R)	0.3	0.1	0.4	0.3	0.7	0.5	0.1	0.3	0.3	0.3	0.3	0.8	0.2	0.1	0.4	0.2	0.9	0.1
Go (L)	0.1	0.2	0.2	0.0	0.3	0.0	0.0	0.6	0.1	0.1	0.8	0.1	0.0	0.6	0.0	0.1	0.6	0.1
Zy (R)	0.1	0.6	0.1	0.4	0.2	0.1	0.2	0.1	0.7	0.1	0.6	0.3	0.2	0.7	0.2	0.3	0.9	0.0
Zy (L)	0.0	0.1	0.3	0.2	0.8	0.1	0.1	0.5	0.2	0.1	0.5	0.5	0.0	0.1	0.4	0.1	0.9	0.3
Mean	0.10	0.11	0.16	0.14	0.14	0.14	0.08	0.13	0.16	0.10	0.16	0.18	0.15	0.09	0.18	0.21	0.20	0.12
SD	0.13	0.12	0.11	0.16	0.21	0.14	0.08	0.16	0.15	0.10	0.23	0.19	0.13	0.18	0.12	0.13	0.30	0.12
Random error (CC)	0.99	0.99	0.99	0.99	0.99	0.99	0.99	0.99	0.99	0.99	0.99	0.99	0.99	0.99	0.99	0.99	0.99	0.99
Systematic error (p-values)	0.389	0.397	0.074	0.143	0.300	0.372	0.477	0.848	0.237	0.888	0.830	0.287	0.929	0.129	0.560	0.193	0.578	0.932

Systematic error was assessed by using a paired t-tests and random error assessed by coefficients of reliability.

## Results

### Topographic analysis

**Figure 4:**
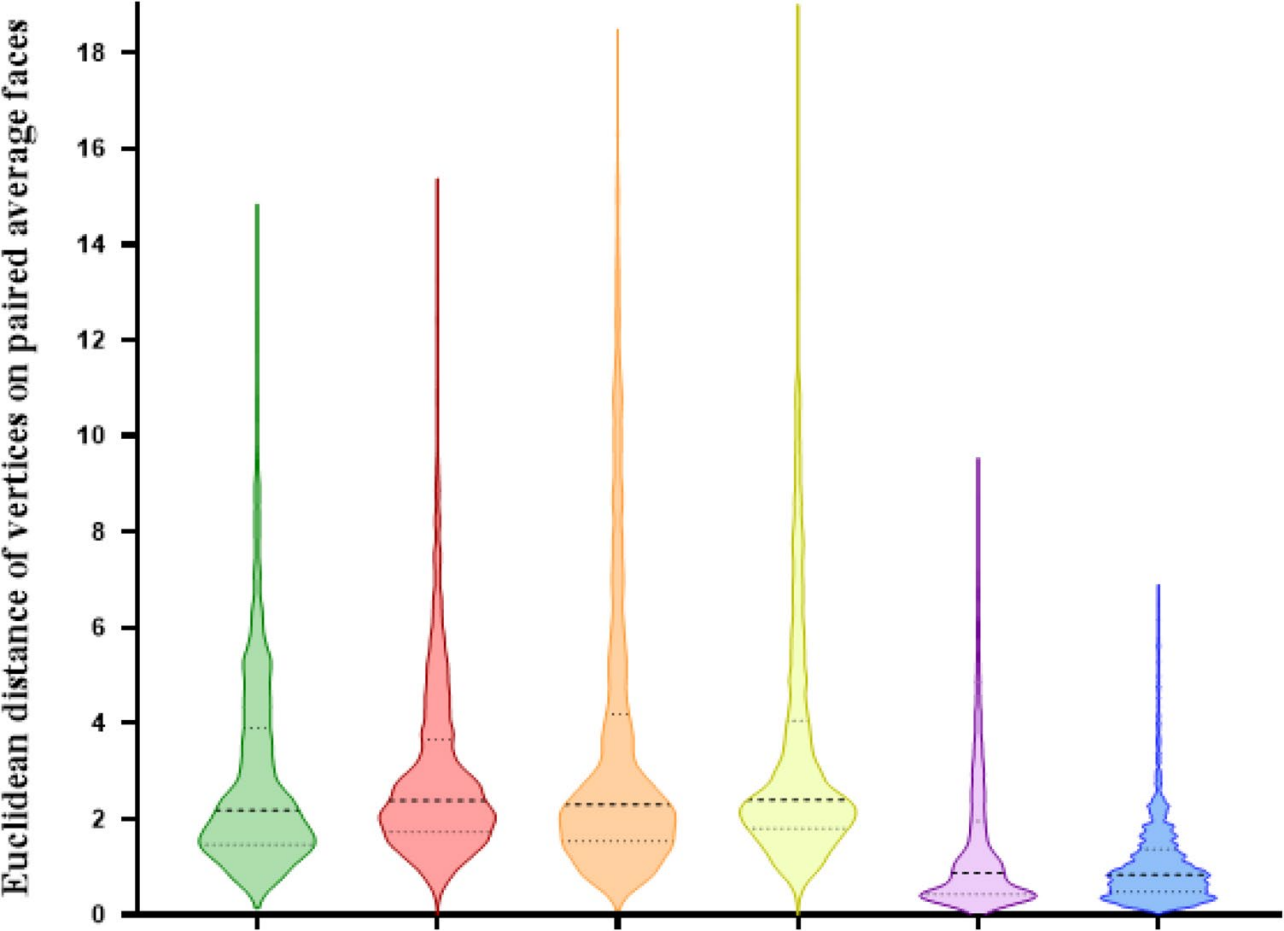
Topographic surface analysis of four average faces generated by Di3DView with 15 (Ave_D15) or 24 landmarks (Ave_D24), and MorphAnalyser with 15 landmarks (Ave_MG15) and 24 landmarks (Ave_MG24) for imaging conformation and construction.

**Figure 5:**
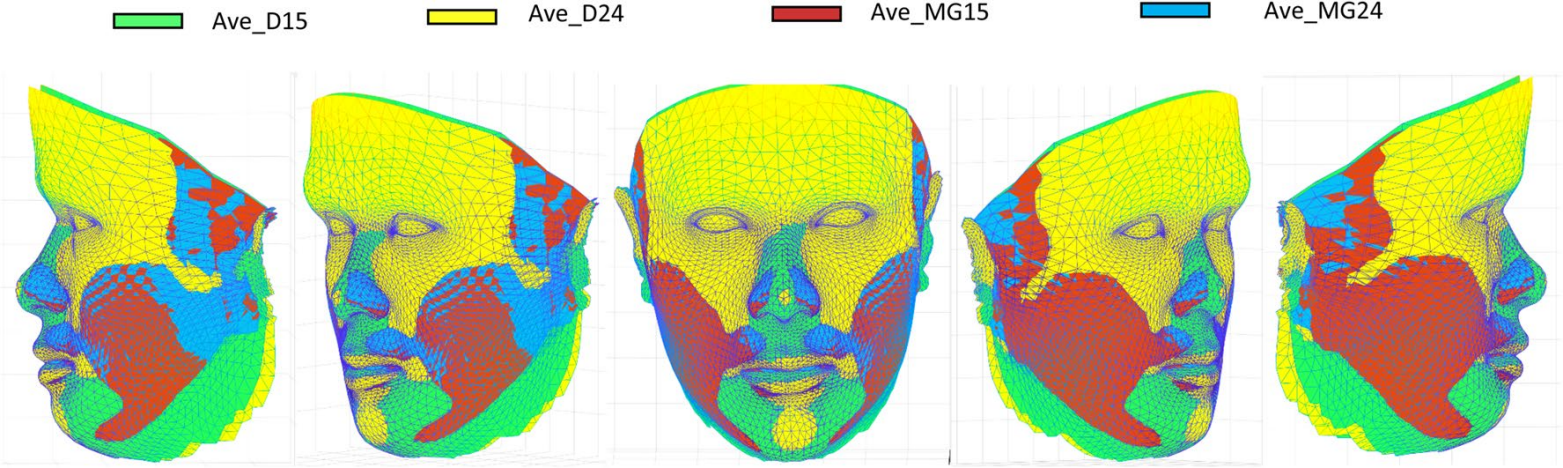
Superimposed average faces, based on the generic mesh and Di3DView (Ave_D15 and Ave_D24), and MorphAnalyser (Ave_MG15 and Ave_MG24).

For the six different combinations of average faces based on the generic mesh (Ave_D15, Ave_D24, Ave_MG15, Ave_MG24) the MED ranged from 0.8 to 2.4 mm. Using the same number of landmarks but different software packages produced some differences in the MED (Ave_D15 & Ave_MG15 = 2.2 mm and Ave_D24 & Ave_MG24 = 2.4 mm). Following a one-sample Wilcoxon signed test, these differences were statically significantly greater than 2.0 mm (p < 0.001), Table 3. In addition, increasing the number of landmarks but using the same software produced minimal changes in the MED (Ave_D15 & Ave_D24 = 0.9 mm and Ave_MG15 & Ave_MG24 = 0.8 mm). Following a one-sample Wilcoxon signed test, these differences were statically significantly less than 2.0 mm (p < 0.001), Fig. 4**.** The superimposed 4 average faces based on the generic mesh and Di3DView and MorphAnalyzer are shown in Fig. 5.

**Table 3:** Topographic surface analysis of three average faces generated by Di3DView with 15 (Ave_D15) or 24 landmarks (Ave_D24), and MorphAnalyser with 15 landmarks (Ave_MG15) and 24 landmarks (Ave_MG24) for imaging conformation and construction.

	Ave_D15 vs Ave_MG15	Ave_D15 vs Ave_MG24	Ave_D24 vs Ave_MG15	Ave_D24 vs Ave_MG24	Ave_D15 vs Ave_D24	Ave_MG15 vs Ave_MG24
Median (mm)	2.2	2.4	2.3	2.4	0.9	0.8
25th percentile (mm)	1.5	1.7	1.5	1.8	0.4	0.5
75th percentile (mm)	3.9	3.7	4.2	4.1	2.0	1.4
One sample Wilcoxon sign test	< 0.001^a^	< 0.001^a^	< 0.001^a^	< 0.001^a^	< 0.001^b^	< 0.001^b^

^a^Statistically greater than 2.0 mm.

^b^Statistically less than 2.0 mm.

### Anthropometric assessment

#### Error study

No systematic errors were observed, all p-values were greater than 0.05. There was no random error, all correlation coefficients are above 0.95. All landmarks were digitised to within 1.0 mm, Table 2.

#### Anthropometric assessment

**Figure 6:**
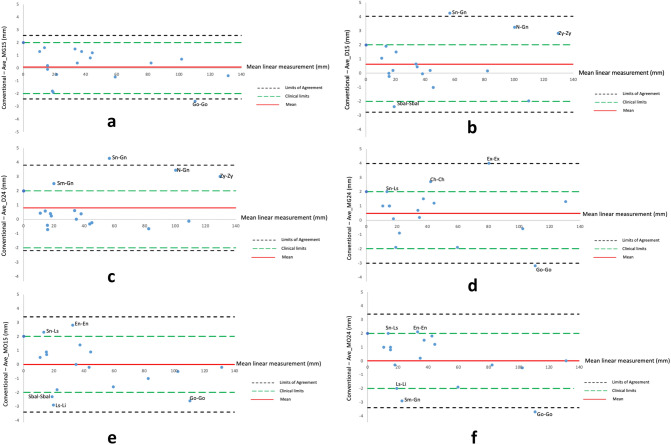
Bland–Altman plots showing agreement between linear measurements obtained from conventional digital anthropometry and from the (**a)** Ave_MG15, (**b)** Ave_D15, (**c)** Ave_D24, (**d)** Ave_MO24, (**e)** Ave_MG24, (**f)** Ave_MO15 respectively.

The anthropometric validity of the four different average faces were evaluated by comparing 17 linear and 7 angular measurements derived from each average face to the gold standard mean measurements. Regarding linear measurements, the Ave_MG15 face showed the highest validity with the least number of parameters over 2 mm (Go-Go: 2.6 mm). The Bland–Altman plot highlights the narrow level of agreement and clinical significance levels, Fig. 6a. Three average faces, Ave_D15, Ave_D24, and Ave_MG24, each had four linear measurements with a difference greater than 2.0 mm. For Ave_D15 and Ave_D24, three parameters were common (N-Gn, Zy-Zy, and Sn-Gn) in addition to Sbal(L) – Sbal(R) (− 2.4 mm) in Ave_D15 and Sm-Gn (2.5 mm) in Ave_D24; for Ave_MG24, clinically-significant differences were observed in Ex(L) – Ex(R) (4.0 mm), Ch(L) – Ch(R) (2.7 mm), Sn-Ls (2.1 mm), and Go(L) – Go(R) (− 3.7 mm), Fig. 6b,c,d. The most errors were seen in Ave_ MO15 and Ave_MO24 with five linear measurements greater than 2.0 mm, Fig. 6e,f. For angular measurements, Ave_MG15, Ave_MO15, and Ave_MO24 showed no parameter with absolute difference over 5 degree. Other average faces, i.e. Ave_D15, Ave_D24, and Ave_MG24 presented a relative inconsistency, particularly for Labiomental angles (− 5.1° to − 10.3°). Additionally, Nasal tip angle and Nasolabial angle in Ave_D15 were also considerably different from the arithmetic mean (6.0° and 9.2° respectively), Table 4. The use of 9 additional landmarks had little effect on the validity of the angular and linear measurements.

**Table 4:** Comparison of parameters in all average faces generated by different imaging systems, different landmark configuration, and different base facial templates.

Parameter	Origin	Ave_D15	Ave_D24	Ave_MG15	Ave_MG24	Ave_MO15	Ave_MO24	Ave_D15 vs origin	Ave_D24 vs origin	Ave_MG15 vs origin	Ave_MG24 vs origin	Ave_MO15 vs origin	Ave_MO24 vs origin	Ave_D15 Ave_D24	Ave_M15_pool_ Ave_M24_pool_	Ave_MG_pool_ Ave_MO_pool_
**Linear**																
En(L)–En(R)	34.0	33.3	33.3	32.5	33.3	31.2	31.9	0.7	0.7	1.5	0.7	2.8	2.1	0.0	−0.7	1.4
Ex(L)–Ex(R)	82.2	82.1	82.9	81.8	78.2	83.2	82.5	0.1	−0.7	0.4	4.0	−1.0	−0.3	-0.8	0.7	−2.8
N–Prn	38.1	38.1	37.7	36.8	36.6	36.7	36.6	0.0	0.4	1.3	1.5	1.4	1.5	0.4	0.1	0.0
Al(L)–Al(R)	34.8	34.3	34.7	34.4	34.6	34.8	34.6	0.5	0.1	0.4	0.2	0.0	0.2	−0.4	0.2	−0.2
Ls–Li	18.3	18.1	18.1	20.2	20.2	21.2	20.3	0.2	0.2	−1.9	−1.9	−2.9	−2.0	0.0	0.9	−0.6
Ch(L)–Ch(R)	43.4	43.2	43.8	42.6	40.7	43.6	41.6	0.2	−0.4	0.8	2.7	−0.2	1.8	−0.6	2.0	−0.9
N–Gn	102.0	98.8	98.6	101.3	102.6	102.5	102.5	3.2	3.4	0.7	-0.6	-0.5	-0.5	0.2	0.0	−0.6
Sbal(R)–Cph(R)	15.5	15.5	15.9	15.6	14.5	14.6	14.7	0.0	−0.4	−0.1	1.0	0.9	0.8	−0.4	−0.1	0.4
Sbal(L)–Cph(L)	15.6	15.8	16.3	15.4	14.6	14.9	14.6	−0.2	−0.7	0.2	1.0	0.7	1.0	−0.5	0.3	0.3
Cph(L)–Cph(R)	11.2	10.1	10.7	9.9	10.2	10.7	10.2	1.1	0.5	1.3	1.0	0.5	1.0	−0.6	0.5	−0.4
Sm–Gn	21.2	19.7	18.7	21.7	22.1	23.0	24.1	1.5	2.5	−0.5	−0.9	−1.8	−2.9	1.0	−1.1	−1.7
Sbal(L)–Sbal(R)	17.8	20.2	17.4	19.6	17.7	20.1	18.1	−2.4	0.4	−1.8	0.1	−2.3	−0.3	2.8	2.1	−0.5
Sn–Ls	14.5	12.6	13.9	12.9	12.5	12.2	12.5	1.9	0.6	1.6	2.1	2.3	2.0	−1.3	−0.3	0.4
Go(L)–Go(R)	108.9	110.9	109.0	111.5	112.1	111.5	112.6	−2.0	-0.1	−2.6	−3.2	−2.6	-3.7	1.9	−1.1	−0.3
Zy(L)–Zy(R)	131.2	128.3	128.1	131.8	129.9	131.4	131.2	2.9	3.1	−0.6	1.3	−0.2	0.0	0.2	0.2	−0.4
N–Sn	44.9	45.9	45.1	43.7	43.7	44.0	43.7	−1.0	-0.2	1.2	1.2	0.9	1.2	0.8	0.3	−0.1
Sn–Gn	58.7	54.5	54.4	59.4	60.6	60.3	60.6	4.2	4.3	-0.7	-1.9	−1.6	−1.9	0.1	-0.3	−0.5
**Angular**																
Nasal root slope	123.5	123.7	125.1	124.8	123.2	124.5	126.1	−0.2	-1.6	−1.3	0.3	−1.0	-2.6	−1.4	−1.6	−1.3
Alar slope angle	80.6	80.4	80.1	81.2	80.5	80.6	80.0	0.2	0.5	−0.6	0.1	0.0	0.6	0.3	0.6	0.5
Labiomental angle	139.3	149.6	145.9	144.2	144.4	141.6	141.9	−10.3	−6.6	−4.9	−5.1	−2.3	−2.6	3.7	−0.3	2.6
N–Sn–Pog	163.9	164.8	168.6	162.8	163.5	163.6	163.5	−0.9	−4.7	1.1	0.4	0.3	0.4	−3.8	0.1	−0.4
N–Prn–Pog	136.2	138.9	138.7	136.2	135.6	136.0	135.6	−2.7	−2.5	0.0	0.6	0.2	0.6	0.2	0.4	0.1
Nasal tip angle	106.8	112.8	108.3	108.5	108.1	108.6	108.0	−6.0	−1.5	−1.7	−1.3	−1.8	−1.2	4.5	0.6	0.0
Nasolabial angle	116.7	125.9	117.6	121.6	120.7	120.3	120.7	−9.2	−0.9	−4.9	−4.0	−3.6	−4.0	8.3	-0.4	0.7

## Discussion

This is the first study to investigate the accuracy of “3D average faces” as an anthropometric tool for facial analysis against conventional methods. With the ever increasing use of digital data and computer software solutions it is important for clinicians to have an understanding of the factors which may affect the output of the software. As clinicians we generally input variables into “blackbox technology/software” and assume the output to be accurate. As with most pieces of conventional laboratory equipment there is an essential step of validation and determining its accuracy; working with digital technology should be no different. The 3D capture systems and software has been validated and their accuracy reported but this does not mean all subsequent applications are as accurate^18, 19^. It is easy to be distracted by the visual superiority of three-dimensional images and just because an image looks “right” it does not mean the image is a true representation of the original image.

Traditional anthropometric measurements based on linear and angular measurements provide measurements of specific areas of the face in a form that is not visually ideal i.e. it does not resemble a face. In addition, the use of Euclidian distances is problematic when measuring 3D distances^20, 21^. The Euclidean distance is by definition the length of a line segment between two points. This means that the distance between two points can be the same but the direction different. For instance, the alar base width using the Euclidian distance and right and left alar landmarks could be the same between cleft and non-cleft individuals. If however the left alar base was depressed in the cleft group, this would only be apparent when differences in the x, y and z position between the right and left alar landmarks were assessed. Therefore moving away from Euclidian distances and using 3D co-ordinates when analysing 3D images would seem more appropriate.

Advances in 3D facial surface imaging have resulted in the ability to capture a human face in 3D as a matter of routine. Recently, a web-based 3D Facial Norms database for European Caucasians has been constructed by synthesizing 2454 individuals covering both genders from 3 to 40 age range^22^. The data contains the 3D coordinates for a variety of standard facial surface landmarks, selected linear distances and face and head measurements using traditional anthropometric methods (i.e. calipers). Interestingly, the 3D images are still analysed as if they were two-dimensional, producing simple linear Euclidian distances and angular measurements. For example, using conventional cephalometery, two points Labiale superius and Stomion (superius) are used to represent the upper lip, but the lip has a complex three-dimensional morphology and is cannot fully described by two landmarks. Three dimensional surface mesh images are made up of vertices, each with a 3D coordinate representing its position in space, in other words each vertex represents a landmark. This means that is possible to represent an anatomical region by the number of vertices it is made up of, with each representing a landmark^23^. Referring back to the previous example of the upper lip, this means that all the vertices making up the lip surface can be utilised. The problem is that each facial 3D image is made of a different number of vertices and therefore there is no consistency between two images, even if they are captured one after the other using the same imaging equipment. To overcome this, the use of a generic mesh and image conformation, or dense correspondence, has been used^24, 25^. Image conformation transforms a generic mesh, made up of a known number of vertices, into the shape of the original 3D facial imaging, whilst maintaining anatomical correspondence between images. This latter feature means that the same vertex (or landmark) between images represents the same anatomical point, e.g. vertex number 345 is always Pogonion point. This then allows for averaging of facial images and the development of “3D average faces”, with each point on the 3D facial mesh acting as a landmark. These landmarks are classified as anatomical, mathematical or pseudo landmarks^26^. Anatomical landmarks are determined by experts and correspond between individuals, mathematical landmarks that located on an object according to some mathematical or geometrical property, i.e. maximum point on a curvature and pseudolandmarks which are constructed points on an object either on the outline or between landmarks. Normal 2D and simple 3D anthropometry use a small number of anatomical landmarks whilst the use of 3D average face allows the use of both anatomical and pseudolandmarks. The additional use of pseudolandmarks allows more detailed measurements. However there is some debate on whether these pseudolandmarks are accurate, with some studies suggesting higher levels of inaccuracies^27, 28^ and others not^15, 29^.

The results of the present study showed that the least differences in anthropometric linear measurements were seen using MorphAnalyzer, the generic mesh and 15 landmarks (Ave_MG15) and was therefore more accurate than the other average faces. The average face generated using Di3DView, the generic mesh and 15 or 24 landmarks for conformation (Ave_D15 and Ave_D24), produced errors upto 4.3 mm (Sn-Gn), compared to the conventional arithmetic means of the individuals. For angular measurements this difference was close to 10° degree for the Labiomental angle using Di3DView, a generic mesh and 15 landmarks for conformation (Ave_D15). For average faces generated using Di3DView, the largest differences were seen in the peripheral region of the face (Gn, Go, and Zy). The addition of further landmarks during the conformation process, from 15 to 24 landmarks, marginally improved the accuracy of the measurements, as seen by the narrower level of agreement, Fig. 6b,c. This was also reflected in the marginal reduction in MED seen by increasing the number of landmarks, using Di3DView and a generic mesh.

**Figure 7:**
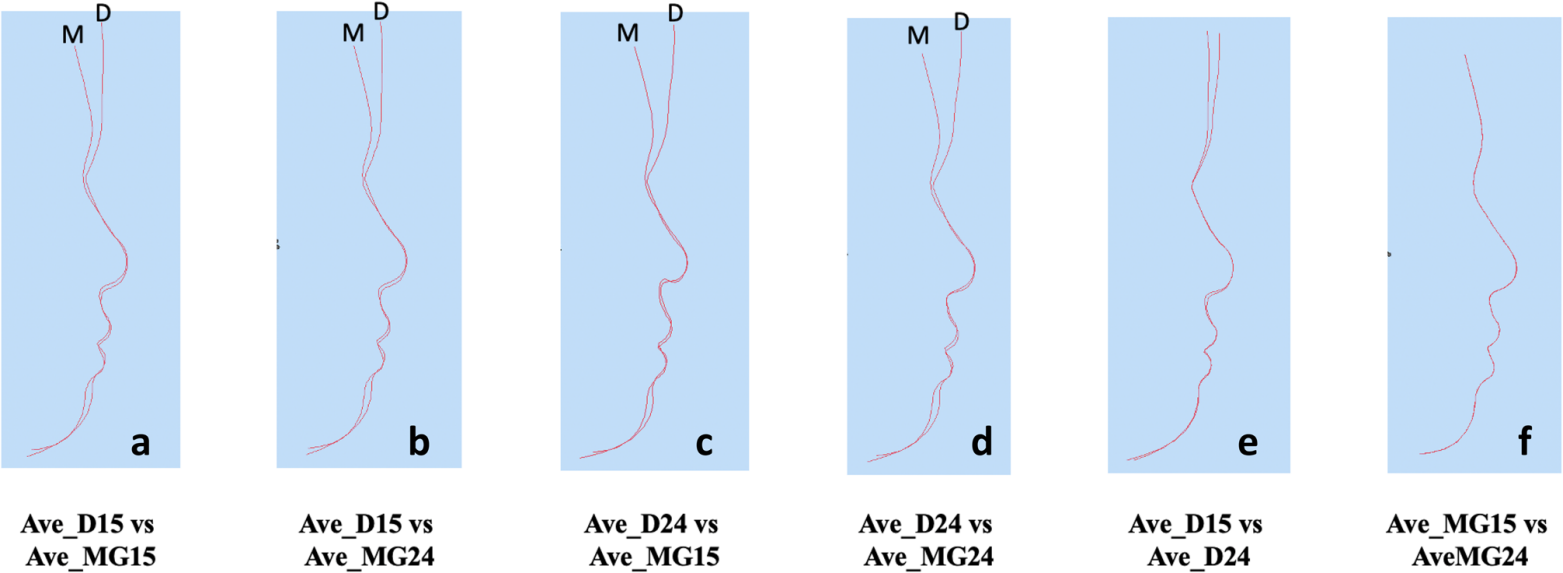
Profile cross-sections between the four different average facial mesh combinations following superimposition on the centroids of each of the average facial meshes. (**a)** Ave_D15 vs Ave_MG15, (**b)** Ave_D15 vs Ave_MG24, (**c)** Ave_D24 vs Ave_MG15, (**d)** Ave_D24 vs Ave_MG24, (**e)** Ave_D15 vs Ave_D24, (**f)** Ave_MG15 vs Ave_MG24.

Based on the topographical analysis all 3D average facial mesh combinations had a MED error greater than 2.0 mm, which would be clinically significant. However this should be viewed with caution, as the MED is the median value of all the points across the 3D entire facial mesh. The discrepancies in the different mesh combinations are not across the entire mesh surface but most marked around the forehead, Fig. 7. These large deviations increase the MED values. There are however small differences in the chin and nasal regions between the different meshes. Interestingly the addition of more landmarks using MorphAnalyser makes little difference to the meshes (Fig. 7f) but has more of an effect on using Di3View (Fig. 7e), again around the forehead region and Subnasle (Sn). This problem has been previously highlighted and the use of regional mesh analysis suggested as a possible solution^30^. The morphology of the forehead is more “realistic” using MorphAnalyser but was not confirmed by anthropometric measurements.

Using Di3DView, additional landmarks improved the accuracy of angular measurements. This was probably due to the additional placement of anatomical landmarks. For instance the addition of Subnasle (Sn) improved the accuracy of the nasiolabial angle. However, this was not the case for the labiomental angle, as Supermentale (Sm) was never an additional landmark, yet additional landmarks improved the accuracy. A possible explanation maybe that the addition of anatomical point Gonion bilaterally may have helped to constrain the mesh during conformation. This highlights the importance of selecting the relevant landmarks and the potential affects they may have on distant landmarks. Measurements involving Gnathian (Gn) using Di3DView produced inaccuracies in the vertical direction i.e. Sn-Gn, N-Gn, Sm-Gn. This is probably a result of incorrect morphology of the chin region produced using Di3Dview and generic mesh and 15 or 24 landmarks, Fig. 2. The error maybe related to the conformation and averaging of peripheral landmarks i.e. Nasion, Gnathian and Zygonion.

For MorphAnalyser the use of the generic mesh rather than an original facial template produces more accurate results. Using the original facial template the intercanthal distance, En(L) – En(R), is inaccurate as well as the vertical lip (Ls-Li & Sn-Ls). The addition of extra landmarks do not improve the general accuracy of the linear measurements. The reason for this is unknown but can only be a result of the software algorithm which produces the average facial mesh. To further complicate the issue the use of a generic mesh improves the anthrometertic accuracy of the measurements; whilst extra landmarks reduces the accuracy. Overall, we could conclude that the validity and accuracy of 3D average faces is dependent upon the software that is being used, the type of baseline / generic mesh and the number of landmarks used during conformation.

## Conclusion

Di3DView and MorphAnalyser are both able to produce 3D average faces for anthropometric analysis. Marginal and non-registered areas were the most inaccurate regions using Di3DView. For MorphAnalyser, the type of template mesh had an effect on the accuracy of the final 3D average face. Additional landmarks did not improve the accuracy. This study highlights the importance of validating software packages and determining the degree of accuracy as well as the variables which may affect the output. The use of MorphAnalyer, a generic mesh and 15 landmarks for conformation produces an 3D average face that that has the same anthropometric dimensions as average measurements determined by conventional anthropometric techniques.
